# Evaluation of oxidative stress biomarkers in patients with chronic renal failure: a case control study

**DOI:** 10.1186/1756-0500-3-20

**Published:** 2010-01-25

**Authors:** Marta Romeu, Rosa Nogues, Luís Marcas, Vanesa Sánchez-Martos, Miquel Mulero, Alberto Martinez-Vea, Jordi Mallol, Montserrat Giralt

**Affiliations:** 1 Pharmacology Unit, Department of Basic Medical Sciences, Universitat Rovira i Virgili, Spain; 2 Nephrology Service, Hospital Universitari de Tarragona Joan XXIII, IISPV, Universitat Rovira i Virgili, Spain; 3 Department of Biochemistry and Biotechnology, Universitat Rovira i Virgili, Spain

## Abstract

**Background:**

Oxidative stress is related to several diseases, including chronic renal insufficiency. The disequilibrium in the oxidant-antioxidant balance is the result of several metabolic changes. The majority of studies to-date have evaluated the grade of oxidative stress with a single biomarker, or a very limited number of them.

**Findings:**

The present study used several important biomarkers to establish a score relating to oxidative stress status (glutathione S-transferase, superoxide dismutase, catalase, glutathione peroxidase, glutathione reductase, reduced and oxidized glutathione, thiobarbituric acid reactive substances and hemolysis test). The score of oxidative stress (SOS) was then applied to a group of patients with renal insufficiency not on hemodialysis, and compared to healthy control individuals.

The score for patients with chronic renal insufficiency was significantly different from that of the healthy control group (0.62 ± 1.41 vs. -0.05 ± 0.94; p < 0.001). The comparison between patients with chronic renal insufficiency and control individuals showed significant differences with respect to changes in the enzymatic antioxidant systems (glutathione S-transferase, glutathione reductase), non-enzymatic antioxidant system (oxidized glutathione) and oxidizability (hemolysis test) indicating significant oxidative stress associated with chronic renal insufficiency.

**Conclusions:**

Patients with chronic renal insufficiency not on hemodialysis are susceptible to oxidative stress. The mechanisms that underlie this status are the consequence of changes in glutathione and related enzymes. The SOS scoring system is a useful biochemical parameter to evaluate the influence of oxidative stress on the clinical status of these patients.

## Findings

Healthy individuals cope with free radicals in the biological system using their own anti-radical "mop-up" systems which quench reactive oxygen species (ROS), scavenge damaged molecules, and repair molecular injuries [1].

There is considerable disequilibrium between oxidants and anti-oxidants in patients with chronic renal insufficiency (CRI). Evidence suggests that ROS are not merely the consequence of treatment or progress of the disease but one of the causal agents of CRI, and that oxidative stress (OS) can take place even in the absence of hemodialysis. Patients with uremia have diminished response to OS due, probably, to a decrease in the antioxidant capacity; the mechanisms underlying this decrease, however, are not well established [2].

In the present study, we propose a score for oxidative stress (SOS) based on the analysis of several biomarkers. Also, we determined the laboratory reference ranges of the SOS parameters in an ostensibly-healthy human population. These markers in the SOS were: glutathione S-transferase (GST), superoxide dismutase (SOD), catalase (CAT), glutathione peroxidase (GPx), glutathione reductase (GR), reduced and oxidized glutathione (GSH, GSSG), thiobarbituric acid reactive substances (TBARS) and hemolysis test (HT). The SOS was applied not only in healthy control individuals but also in patients with CRI.

## Methods

The analyses were conducted in three groups of individuals: Group C1: healthy controls (n = 164) recruited from among the staff of our School of Medicine (age: 48.4 ± 14.5 years; 52 males and 112 females). The individuals in the C1 group were not on any prescribed medications (including anti-oxidants), no subject was an active smoker, the individuals did not participate in regular intense exercise, and all had moderate exposure to the sun; Group CRI: patients with uremia pre-dialysis (n = 63) were recruited from the *Joan XXIII *University Hospital, Tarragona (age: 62.1 ± 14.3 years; 33 males and 30 females). The CRI group comprised a variety of disease etiologies, and with creatinine clearance between 7.7 and 35.8 ml/min [see Additional file 1 - Table S1 for general characteristics of CRI patients]; Group C2: healthy control subjects (n = 63) composed of individuals in the C1 group but age- and gender-matched for those in the CRI group. All participants gave fully informed consent to participation in the study and ethical approval was obtained from the Ethical Committee for Clinical Investigation (*Sant Joan *University Hospital, Reus).

Blood was obtained by venepuncture and collected in lithium-heparin (Li-Hep) as well as EDTA tubes.

### Li-Hep sample

Hematocrit and hemoglobin values were obtained using whole blood. Erythrocytes were preserved at 20°C for subsequent batched GST activity and HT analysis. To determine erythrocyte and plasma GSH and GSSG, and erythrocyte TBARS, samples were deproteinized with cold 70% trichloroacetic acid (TCA) (final concentration 10%) and centrifuged. The TCA acts as a preservative by maintaining the sample in an acid medium and, as such, preempting auto-oxidation. Aliquots of the supernatant were stored at -20°C for subsequent batched analyses.

### EDTA sample

Erythrocytes were stored at -20°C for subsequent batched analysis of SOD, GPx, GR and CAT. Aliquots of plasma were stored at -20°C for subsequent TBARS measurement.

GST activity was calculated by the Habig et al method [3]. We determined GST activity (T-GST) and the thermo-stable GST fraction (TS-GST). The TS-GST fraction was also expressed as the % of T-GST, and termed "residual GST" (%TS-GST). 1-chloro-2,4-dinitrobenzene (CDNB) was used as substrate for GST.

SOD enzyme activity was measured by the Misra and Fridovich method [4] based on the auto-oxidation of epinephrine.

CAT activity was determined by the Cohen et al method [5]. The rate of hydrogen peroxide breakdown was monitored.

GR and GPx activities were determined by the Wheeler et al method [6] which monitors the rate at which NADP^+ ^or NADPH is converted.

GSH and GSSG were determined by fluorimetry using the Hissin and Hilf method using o-phthaldialdehyde (OPT) as the fluorescent reagent [7] and, from which, the GSSG/GSH ratio was calculated.

TBARS were determined by the Buege and Aust method but using fluorescence [8,9]. Lipid peroxidation was measured as malondialdeyde (MDA) equivalents using trichloroacetic acid, thiobarbituric acid and hydrogen chloride.

The hemolysis test was performed according to the Farrell et al method [10] using hydrogen peroxide as hemolytic agent in washed erythrocytes.

To obtain the overall SOS, each parameter received a numerical value (zero, -1 or +1). The laboratory reference ranges were obtained from Group C1. Results within the laboratory reference ranges were assigned zero points (0); results above or below the laboratory reference range, and indicating OS, were assigned one positive point (+1). Results indicating anti-oxidant status (AS) were assigned one negative point (-1) (Table 1).

**Table 1:** Scoring criteria for each biomarker to derive the score of oxidative stress (SOS)

*Biomarkers*	Value > ULN	Value < LLN
**Antioxidant enzymes**		
T-GST	-1	+1
TS-GST	-1	+1
%TS-GST	-1	+1
SOD -	1	+1
CAT	-1	+1
GR	-1	+1
GPx	-1	+1
**GSH, GSSH, GSSG/GSH**		
GSH erythrocytes	-1	+1
GSSG erythrocytes	+1	-1
GSSG/GSH erythrocytes	+1	-1
GSH plasma	-1	+1
GSSG plasma	+1	-1
GSSG/GSH plasma	+1	-1
**Lipid peroxidation products**		
TBARS erythrocytes	+1	-1
TBARS plasma	+1	-1
**Oxidisability measurements**		
HT	+1	-1

-1: antioxidant status; +1: oxidative stress; 0: antioxidant pro-oxidant equilibrium; ULN: upper limit of normal; LLN: lower limit of normal

The data were processed with the SPSS statistical package. The 2.5^th ^and 97.5^th ^percentiles were obtained for all biomarkers in the healthy control population sample and used as the lower and upper limits of "normality" for the patient population. Relationships between the variables were evaluated with the Pearson linear correlation coefficient (r). Multiple linear regression analysis was used to identify predictors of the model. Results from CRI patients and those from C2 healthy controls were compared using the Student *t*-test, or the Mann-Whitney test.

Discriminant analysis was used to statistically evaluate the variables that would distinguish healthy individuals from those with CRI, using the variables of oxidative stress. The results of the discriminant score were compared with the SOS. Statistical significance was set at p < 0.05.

## Results

Table 2 summarizes the values of OS biomarkers in erythrocytes and plasma in healthy control group C1. These are the values we used as the reference limits to calculate SOS. The C1 control group comprises individuals who have not been exposed to the main exogenous factors of ROS production. As such, the values of biomarkers could be used as reference for other OS studies.

**Table 2:** Oxidative stress biomarkers in the C1 group (healthy controls)

Biomarkers of C1	Mean ± SD	LLN-ULN
*Erythrocytes*		
T-GST (μmol/min/g Hb)	1.60 ± 0.48 (163)	0.79-2.63 (163)
TS-GST (μmol/min/g Hb)	0.38 ± 0.24 (146)	0.08-1.00 (146)
%TS-GST	24.33 ± 14.12 (145)	6.44-63.24 (145)
GSH (μmol/g Hb)	5.11 ± 1.48 (164)	2.79-8.51 (164)
GSSG (μmol/g Hb)	0.79 ± 0.42 (164)	0.21-1.98 (164)
GSSG/GSH	0.17 ± 0.11 (164)	0.03-0.39 (164)
TBARS (nmol/g Hb)	4.73 ± 3.01 (158)	1.20-12.92 (158)
CAT (mmol/min/g Hb)	222 ± 38 (149)	144-295 (149)
GPx (μmol/min/g Hb)	28.25 ± 8.10 (133)	13.71-49.74 (133)
GR (μmol/min/g Hb)	3.48 ± 1.38 (149)	1.55-7.57 (149)
SOD (U/g Hb)	1763 ± 535 (155)	914-2806 (155)
HT (%)	12.19 ± 4.83 (150)	5.06-21.57 (150)
*Plasma*		
GSH (nmol/ml)	22.48 ± 12.01 (164)	4.47-50.79 (164)
GSSG (nmol/ml)	24.58 ± 7.39 (164)	11.94-40.09 (164)
GSSG/GSH	1.60 ± 1.32 (164)	0.28-5.27 (164)
TBARS (nmol/ml)	1.86 ± 1.11 (163)	0.30-4.76 (163)

Reference ranges and means ± SD (n) for the biomarkers of oxidative stress where "n" is the number of subjects per group. The lower limit of normal (LLN) and the upper limit of normal (ULN) are the 2.5th and 97.5th percentiles, respectively.

Table 3 summarizes the significant Pearson correlations between biomarkers. There were several correlations:

**Table 3:** Significant Pearson correlation coefficients between oxidative stress biomarkers in the C1 group

Biomarkers	PC	Biomarkers	PC	Biomarkers	PC
TBARS p - TBARS e	0.358	TBARS e -GSH p	0.184	T-GST -TBARS p	-0.186
TBARS p - GSH e	-0.292	TBARS e -GSSG/GSH p	-0.227	T-GST -GSH e	0.205
TBARS p - GSSG e	-0.218	TBARS e -T-GST	-0.188	T-GST -GSH p	-0.157
TBARS p - GSSG/GSH p	-0.176	TBARS e -TS-GST	-0.173	T-GST -GSSG/GSH p	0.219
TBARS p - GR	0.367	TBARS e -SOD	-0.292	T-GST -TS-GST	0.446
GSSG e - GSSG/GSH e	0.866	GSSG/GSH e -GSH e	-0.45	TS-GST -TBARS p	0.224
GSSG e - T-GST	0.213	GSSG/GSH e -%TS-GST	-0.235	TS-GST -GSSG e	-0.189
GSSG e - SOD	-0.206	GSSG/GSH e -SOD	-0.161	TS-GST -%TS-GST	0.805
GSSG e - CAT	-0.194	GSSG/GSH e -GR	-0.161	TS-GST -GR	0.303
GSSG e - GR	-0.189				
GSH p - GSSG p	-0.202	GSSG p -GSSG e	0,390	%TS-GST -TBARS p	0.342
GSH p - GSSG/GSH p	-0.725	GSSG p -GSSG/GSH e	0,373	%TS-GST -GSSG e	-0.325
GSH p - %TS-GST	-0.167	GSSG p -GSSG/GSH p	0,379	%TS-GST -GR	0.341
GSH p - GR	-0.195	GSSG p -SOD	-0,197	%TS-GST -GPx	-0.299
CAT - TBARS p	0.205	GPx -TBARS p	-0.377		
CAT - GSH e	0.193	GPx -GSH e	0.196		
CAT - GSSG/GSH e	-0.268	GPx -GSSG e	0.185		
CAT - GR	0.218	GPx -HT	0.182		
CAT - HT	-0.258				

Pearson correlation (PC) for the biomarkers of oxidative stress; "e" and "p" are measurements performed in erythrocytes and plasma, respectively

#1: between peroxidation products and antioxidant enzymes [i.e. erythrocyte TBARS and SOD (r = -0.292; *p *< 0.001), erythrocyte GSSG and SOD (r = -0.201; *p *= 0.01), erythrocyte GSSG and GR (r = -0.189; *p *= 0.021) and erythrocyte GSSG and GPx (r = 0.185; *p *= 0.033)].

#2: between oxidizability and antioxidant enzymes [i.e. HT and GPx (r = 0.182; *p *= 0.036)].

#3: between lipid and peptide peroxidation products.

#4: between antioxidant enzymes and low-molecular-weight antioxidants [i.e. T-GST and erythrocyte GSH (r = 0.205; *p *= 0.009)].

#5: correlations within the same category [i.e. erythrocyte and plasma TBARS (r = 0.358; *p *< 0.001), erythrocyte GSSG and GSSG/GSH (r = 0.866, *p *< 0.001) and TS-GST and %TS-GST (r = 0.805, *p *< 0.001)].

The larger number of statistically significant correlations indicates that assessing OS using a single biomarker is unacceptable. Based on previous studies [11], and the current data, we believe that joint evaluation of several biomarkers would provide more realistic, and comprehensive, data on antioxidant *versus *pro-oxidant balance.

Some predictor biomarkers were found by multiple linear regression analysis (R^2^). Most biomarkers can be predicted by other, interrelated, biomarkers. Those with the highest values of R^2 ^are shown in Table 4. Some studies have suggested that the ratio of SOD/(GPx + CAT) activities in erythrocytes, rather than the absolute amounts of individual antioxidant enzymes, is indicative of oxidative imbalance [12]. Other studies propose a mathematical model of glutathione metabolism as a tool to investigate the influence of oxidative stress on some pathologies [13].

**Table 4:** Predictor variables identified by multiple linear regression analysis in the C1 group data

Biomarker			Biomarker			Biomarker		
Dependent	Predictor	R^2^	Dependent	Predictor	R^2^	Dependent	Predictor	R^2^
**TS-GST**	%TS-GST	0.927	**TBARS p**	%TS-GST	0.538	**GSH e**	GSSG/GSHe	0.716
	T-GST			TBARS e			GSSG e	
	TBARS p			GR			TBARS p	
	GSH p			GPx			GSSG p	
				GSH e				
**%TS-GST**	TS-GST	0.913	**TBARS e**	TBARS p	0.261	**GSSG/GSH p**	GSH p	0.681
	T-GST			TS-GST			GSSG p	
	TBARS p			GSSG/GSH e			TBARS e	
	GSH p			SOD				
**T-GST**	TS-GST	0.775				**GSH p**	GSSG/GSH p	0.624
	%TS-GST						GR	
	GSH p						GSSG e	
**GPx**	TBARS p	0.223	**GR**	TBARS p	0.248	**GSSG p**	GSSG/GSH p	0.382
	CAT			GPx			GSSG e	
	HT						TBARS e	

Correlation between the observed and predicted values of the dependent variable, multiple correlation coefficient (R2) for the biomarkers of oxidative stress. Multiple linear regression analysis using the "stepwise" procedure; "e" and "p" are measurements performed in erythrocytes and plasma, respectively

The weighting of each biomarker in the scoring system proposed in this article (Table 1) was derived from data from the literature and from the statistical analyses of the current results from group C1.

The values of the antioxidant enzymes GST, SOD, CAT, GPx and GR were scored in a similar manner. High values of antioxidant enzymes therefore indicate antioxidative status (AS score: -1) and low values indicate oxidative stress (OS score: +1). An increase in TBARS content was taken as indicative of oxidative damage. Therefore, TBARS values above the upper limit of normal (ULN) were assigned +1 point (OS) in plasma as well as erythrocytes because of the positive correlation between them.

The GSSG/GSH ratio is considered the redox value that best determines the antioxidant capacity of cells [14], and any increase suggests a strong pro-oxidant effect [15]. In the SOS, high values of both the GSSG and the GSSG/GSH ratio indicate OS (score: +1) and low values indicate AS (score: -1). GSH is an important low-molecular-weight antioxidant and, as such, high values would indicate AS (score -1) and low values would indicate OS (score +1).

The validity of GSH and TBARS as biomarkers has been criticized [16,17]. Further, the values in plasma can be modified in the course of obtaining the sample i.e. oxidation of the molecule can occur as a result of the action of the endogenous enzyme present in plasma. For this reason, although the necessary conservatory precautions were taken to minimize this occurrence, the biomarkers GSH, GSSG and TBARS in plasma may be eliminated from the group of biomarkers comprising the SOS.

HT is an indicator of the susceptibility of erythrocytes to OS. A high HT was therefore considered to be indicative of OS (score: +1) and low levels to be indicative of AS (score: -1).

**Figure 1:**
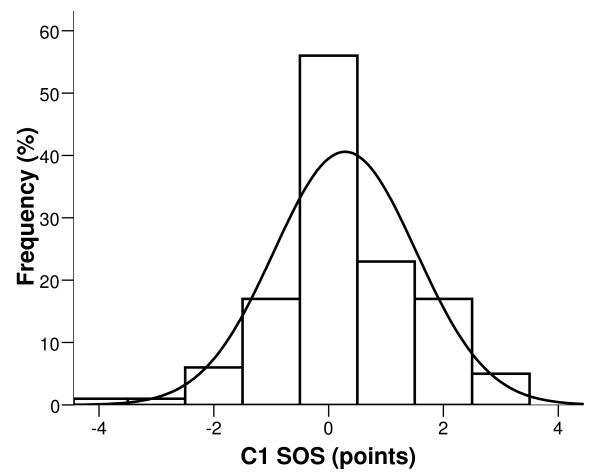
**Score of oxidative stress in a healthy group of individuals**. Frequency distribution of score of oxidative stress (SOS) in the C1 control group (bars). The normal distribution curve is superimposed on the histogram.

Finally, the new multi-biomarker score (SOS) was obtained using a simple mathematical formula (SUM function) linked to a Microsoft Excel^® ^spreadsheet. The SOS values in the C1 group were normally distributed around the mean which, by definition, was 0 points (Figure 1).

**Figure 2:**
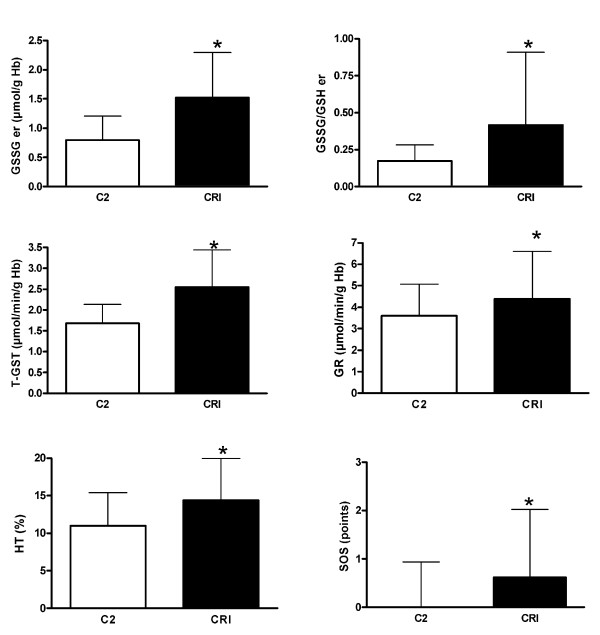
**Oxidative stress biomarkers in patients with uremia**. Biomarkers of oxidative stress and score of oxidative stress (SOS) in the C2 control group, and in the pre-dialysis patients with uremia (CRI group) (mean ± SD). * p < 0.05 for CRI versus C2.

Figure 2 shows the significant differences in the biomarkers between the C2 and the CRI groups.

Changes in the enzymatic antioxidant systems (GST, GR), non-enzymatic antioxidant system (GSSG) and oxidizability (HT) indicate OS in CRI patients. Erythrocyte GSSG and GSSG/GSH were higher in CRI patients than in C2 subjects (1.52 ± 0.77 vs. 0.80 ± 0.41, *p *< 0.001; 0.42 ± 0.49 vs. 0.17 ± 0.11, *p *< 0.001), as were T-GST (2.55 ± 0.89 vs. 1.68 ± 0.45, *p *< 0.001) and GR (4.39 ± 2.22 vs. 3.60 ± 1.47, *p = *0.001) enzymes. HT was also significantly higher in CRI patients (14.44 ± 5.48 vs. 11.08 ± 4.41, *p *< 0.001). Previous studies have shown higher GSSG levels in patients with uremia, together with increased activities of GST and GR in erythrocytes [14]. Also, there have been studies in which the GPx activity in patients was lower than in healthy group, although the decrease becomes more marked when the GPx activity is measured in plasma than in red blood cells [18,19]. In our case, the levels of GPx were measured in erythrocytes and the results are similar in both groups. In our study, if each biomarker is considered individually, there appears to be no clear distinction between C2 and CRI individuals, especially in relation to some of the biomarkers such as TBARS, SOD, CAT [see Additional file 2 - Table S2 for oxidative stress biomarkers in the C2 and CRI groups]. Nevertheless, SOS was significantly higher in CRI patients (0.62 ± 1.41 vs. -0.05 ± 0.94, *p *< 0.001) which suggests that SOS could be a useful multi-biomarker of clinical OS. A reliable measure of the global anti-oxidant capacity in CRI patients would be useful but, unlike in other diseases, the determination of a total antioxidant potential (i.e. by measuring the ability of a biological fluid to resist oxidation) failed to act as a reliable parameter [20].

We used the discriminant analysis to evaluate the "manual" scoring [21]. The discriminant statistical analyses derived an equation that generates a score for each individual. The discriminant score is used to enhance the separation between the individuals in the C2 and CRI groups.

The variables that the statistical discriminant test used to construct the equation, and to assign a score to each individual, were: T-GST, TS-GST, erythrocyte and plasma GSSG/GSH and HT.

A total of 92.1% of individuals in the C2 group and 82.5% in the CRI group were classified correctly using the discriminant score.

SOS correlated positively with the discriminant score (R = 0.243, *p *= 0.006). As such, the SOS, as well as the discriminant analysis score, provides quantitative measures of OS. However, discriminant analysis has some disadvantages, the Fisher discriminant function derived from the two groups in the current study may not apply to other groups of patients with different pathologies (a group of patients with respiratory disease, for example).

In conclusion, the most important mechanism that causes disequilibrium between oxidants and antioxidants in patients with CRI is, essentially, glutathione oxidation, and the related enzymes. Further, the susceptibility of the erythrocyte membranes to oxidation is increased in these patients.

Interestingly, when we used the SOS system we noted increased OS in the CRI population, even in those who were not on dialysis. Therefore, our multi-biomarker could provide a reliable new biochemical parameter (SOS) to evaluate the clinical status of these patients.

## List of abbreviations

ROS: Reactive oxygen species; OS: Oxidative stress; AS: Antioxidant status; SOS: Score for oxidative stress; CRI: Chronic renal insufficiency; GSH: Reduced glutathione; GSSG: Oxidized glutathione; GST: GSH S-transferase; SOD: Superoxide dismutase; CAT: Catalase; GR: GSH-reductase; GPx: GSH-peroxidase; TBARS: Thiobarbituric acid reactive substances; HT: Hemolysis test; TCA: Trichloroacetic acid; MDA: Malondialdeyde.

## Competing interests

The authors declare that they have no competing interests.

## Authors' contributions

MR was involved in the study design, sample collection, analysis and interpretation of the data, and in writing of the report; MG and RN participated in the sample collection, analysis and interpretation of the data, and in drafting the manuscript; LM, VS and MM contributed to data collection and analysis; AM and JM participated in design and coordination of the study, and contributed to the drafting and reviewing of the manuscript.

All authors have read and approved the final manuscript.

## Supplementary Material

Additional file 1 **General characteristics of CRI patients (n = 63)**. Distribution of clinical and biochemical variables Click here for file

Additional file 2 **Oxidative stress biomarkers in the C2 and CRI groups**. Means ± SD for the biomarkers of oxidative stress in the C2 control group, and in the pre-dialysis patients with uremia (CRI group), where "n" is the number of subjects per group. * p < 0.05 for CRI versus C2 Click here for file
