# Supplementary material for: Evaluation of oxidative stress biomarkers in patients with chronic renal failure: a case control study
Source: BMC Res Notes. 2010 Jan 25;3:20. doi: 10.1186/1756-0500-3-20 (PMC2843731; doi:10.1186/1756-0500-3-20)
Supplement: Additional file 1 — General characteristics of CRI patients (n = 63). Distribution of clinical and biochemical variables [file 1756-0500-3-20-S1.PDF]

STable 1: General characteristics of CRI patients (n=63)

Gender	
Males	52.4 %
Females	47.6 %
Age	62.1 ± 14.3 years
Etiologies	
Diabetes	17.5 %
Vascular	19.0 %
Glomerular	19.0 %
Tubulo-interstitial	28.6 %
Unknown	15.9 %
Non Smokers	84.1 %
Other diseases	
Cardiac insufficiency	11.1 %
Arrhythmia	6.3 %
Ischemic cardiopathy	3.2 %
Cardiac surgery/angioplasty	11.1 %
Diabetes mellitus	17.5 %
Arterial hypertension	82.5 %
Clinical and biochemical parameters	
Creatinine clearance (Cochcroft-Gault formula)	17.46 ± 7.03
Cardiac frequency (beats/min)	74.10 ± 12.01
Body mass index	27.35 ± 5.36
Calcium (mg/dL)	2.29 ± 0.22
Phosphorus (mg/dL)	1.65 ± 0.36
Alkaline phosphatase (U/L)	199.71 ±107.6
Albumin (g/L)	36.80 ± 6.29
Hemoglobin (mg/dL)	9.93 ± 0.69
Transferrin saturation (%)	28.71 ± 13.85

Distribution of clinical and biochemical variables
