# Supplementary material for: Evaluation of oxidative stress biomarkers in patients with chronic renal failure: a case control study
Source: BMC Res Notes. 2010 Jan 25;3:20. doi: 10.1186/1756-0500-3-20 (PMC2843731; doi:10.1186/1756-0500-3-20)
Supplement: Additional file 2 — Oxidative stress biomarkers in the C2 and CRI groups. Means ± SD for the biomarkers of oxidative stress in the C2 control group, and in the pre-dialysis patients with uremia (CRI group), where "n" is the number of subjects per group. * p < 0.05 for CRI versus C2 [file 1756-0500-3-20-S2.PDF]

STable 2: Oxidative stress biomarkers in the C2 and CRI groups

	C2 (n=63)	CRI (n=63)
	Mean ± SD	Mean ± SD
Erythrocytes		
T-GST (μmol/min/gHb)	1.68 ± 0.45	2.55 ± 0.89 *
TS-GST (μmol/min/gHb)	0.38 ±. 0.22	0.39 ± 0.34
%TS-GST	22.33 ± 10.86	16.45 ± 14.74
GSH (μmol/g Hb)	5.18 ± 1.57	4.95 ± 2.26
GSSG (μmol/g Hb)	0.80 ± 0.41	1.52 ± 0.77 *
GSSG/GSH	0.17 ± 0.11	0.42 ± 0.49 *
TBARS (nmol/g Hb)	4.96 ± 2.84	5.15 ± 5.48
CAT (mmol/min/g Hb)	226 ± 33.6	229 ± 100.8
GPx (μmol/min/g Hb)	27.49 ± 7.39	29.83 ± 8.86
GR (μmol/min/g Hb)	3.60 ± 1.47	4.39 ± 2.22 *
SOD (U/g Hb)	1676 ± 474	1632 ± 694
HT (%)	11.08 ± 4.41	14.44 ± 5.48 *
Plasma		
GSH (nmol/ml)	23.31 ± 13.36	28.84 ± 15.55
GSSG (nmol/ml)	25.28 ± 7.77	31.58 ± 15.55
GSSG/GSH	1.67 ± 1.42	1.47 ± 1.34
TBARS (nmol/ml)	2.05 ± 1.11	2.16 ± 0.82

Means ± SD for the biomarkers of oxidative stress in the C2 control group, and in the pre-dialysis patients with uremia (CRI group), where “n” is the number of subjects per group. * $p<0.05$ for CRI versus C2
